# Utilizing patient advocates in Parkinson’s disease: A proposed framework for patient engagement and the modern metrics that can determine its success

**DOI:** 10.1111/hex.13064

**Published:** 2020-05-03

**Authors:** Megan Feeney, Christiana Evers, Danielle Agpalo, Lisa Cone, Jori Fleisher, Karlin Schroeder

**Affiliations:** ^1^ Parkinson’s Foundation New York New York; ^2^ Section of Movement Disorders Department of Neurological Sciences Rush University Medical Center Chicago Illinois

**Keywords:** methodology, metrics, patient advocacy, patient empowerment, patient engagement, patient involvement

## Abstract

The wide application of patient engagement and its associated benefits has increased across government, academic and pharmaceutical research. However, neither an identified standard practice for the process of engagement, nor utilization of common metrics to assess associated outcomes, exists. Parkinson's Foundation developed a patient engagement framework and metrics to assess engagement within the academic research and drug development sectors. This approach was developed over the course of several years through assessing the literature, acquiring feedback from researchers and people with Parkinson's disease and adapting practices to be relevant and generalizable across patient engagement projects. This framework includes the: 1) creation of a scope of work, 2) establishment of guiding principles, 3) selection and training of participants, 4) co‐determination of project metrics, 5) execution of the project and 6) dissemination of project findings. Parkinson's Foundation has also worked with academic, government and pharmaceutical stakeholders to identify metrics that assess both the quality of patient engagement and outcomes associated with patient engagement on projects. By improving patient engagement project methodologies and metrics, global clinical trials can have access to evidence‐based patient engagement practices to more efficiently capture the needs of, and potentially benefit, the patient community.

## 1 INTRODUCTION

Residing within the umbrella of community‐based participatory research, patient engagement has created a paradigm shift for many traditional, medical research methods. Beginning as an effort to expedite the availability of new drugs to the public, the Food and Drug Administration (FDA) first recognized patient involvement in the late 1980s with the creation of HIV patient advocacy groups.^1^ In the last four decades, patient involvement in research, commonly known as patient engagement, has transitioned from advocacy to active engagement throughout each stage of the research process, with patients owning a critical role in determining the direction and outcomes of research.

Patient engagement in research gained further attention in 2010, with the passing of the Affordable Care Act (ACA), in which Congress authorized the creation of the Patient‐Centered Outcomes Research Institute (PCORI), an independent, non‐profit, non‐governmental organization.^2^ PCORI’s mission requires the active involvement of patients, caregivers and the broader health‐care community in studies that can help patients make informed health‐care decisions and improve health‐care delivery outcomes.^2^ An analysis of the projects funded through PCORI have suggested that patient engagement results in research that is better aligned with patient and physician needs, including research question relevance, recruitment and retention of study participants, data collection processes, interpretation of results and dissemination.^3^

In 2012, with the authorization of the fifth Prescription Drug User Fee Act (PDUFA V), the FDA created the Patient‐Focused Drug Development Initiative (PFDD) to expand engagement beyond academic research. PFDD hosted a series of meetings between 2012 and 2017 to systematically acquire patient perspectives, needs and priorities on specific diseases (including Parkinson's disease) and their affiliated treatments, for the purpose of integrating these findings into drug development and evaluation.^4^, ^5^, ^6^ In addition to collecting patient insights and preferences, PFDD meetings were also held to identify and facilitate the use of robust methods and best practices to collect the information most important to patients.^5^ PFDD Draft Guidance 1 was released in 2018, and Guidance 2‐4 are scheduled to be released in the next several years.^1^

The wide application of patient engagement and its associated benefits has increased the practice across government, academic and pharmaceutical research. However, even with its increasing application,^7^ neither an identified standard practice for the process of engagement, nor utilization of common metrics to assess the outcomes, exists.^8^ Currently, engagement and input can range from minimal activity to complex involvement.^7^, ^9^, ^10^ In examples of minimal engagement, organization agendas are often set and defined prior to involvement, and patients have limited abilities to influence research and drug development.^9^ In contrast, more active and purposeful engagement in research considers patients to have equal decision‐making authority. In this higher end of the spectrum, patients work with researchers to identify priorities, set agendas and design trials.^9^

Several interdisciplinary health organizations have worked in partnership to develop generalizable recommendations to foster positive patient engagement practices. Many of the deliverables developed through these partnerships are publicly available for use. The affiliated groups include Patient‐Focused Medicines Development, the Clinical Trials Transformation Initiative, the aforementioned PCORI, Drug Information Association and European Patients’ Academy. These organizations along with patient advocacy organizations, including Parkinson's Foundation, have led the charge in identifying, practicing and refining the foundations of patient engagement.

The Parkinson's Foundation determined that there was an urgent need for patient engagement in Parkinson's research following the work of the HIV/AIDS and breast cancer community. Patient engagement was critical in those disease communities, not only because survival rates were poor, but also because heterogeneity of disease and complex symptoms meant that a wide range of patient experience needed to be accounted for to design optimal trials. Similarly, Parkinson's disease (PD) has a wide heterogeneity of disease and complex symptom set. PD is a progressive neurological disorder affecting nearly ten million people worldwide.^11^ It is characterized by motor symptoms, including slowed movement, balance and gait difficulties, rigidity and tremors. In addition, people with PD experience non‐motor symptoms such as cognitive impairment, gastrointestinal issues, anxiety and depression.^11^ No two people with PD experience the same set of symptoms in the same order, or with the same rate of progression.

While heterogeneity of disease and complex symptoms highlight the critical need for patient engagement, they can also make patient engagement more challenging by introducing multiple variables. A consistent approach to patient engagement that encompasses potential variables becomes crucial to ensuring patients can meaningfully engage in the research process. This, in addition to the increasing expectation of the incorporation of patient engagement in the research and drug development process by both the FDA^12^, ^13^ and patient advocacy groups, highlights the need to standardize patient engagement frameworks and methodologies and assess the affiliated outcomes through metrics. Parkinson's Foundation supports the standardization of methods and metrics that promote a high level of engagement and defines patient engagement as the inclusion of patients as equal partners in research decision making at each step of the research or drug development process. This article presents a Parkinson's Foundation‐developed patient engagement framework and metrics to assess such engagement within the academic research and drug development sectors. The Foundation has a long history of sharing tools, resources and best practices with different disease communities, and the Foundation's models have proven adaptable and replicable in other disease areas. By standardizing patient engagement methods, patient engagement activities can be validated, refined and reproduced. With the identification of evidence‐based practices, the value of effective patient engagement practices can be justified and widely replicated.

## 2 A PROPOSED FRAMEWORK FOR PATIENT ENGAGEMENT

Parkinson's Foundation developed its patient engagement model in 2008 with the Parkinson's Advocates in Research (PAIR) programme. Since then, more than 350 people with PD and care partners have been trained in the academic research and drug development process. The PAIR programme trains Research Advocates to prioritize research (eg serve on advisory boards, provide input on therapeutic targets), improve studies (eg enhance study protocols, ensure impactful outcomes) and influence stakeholders (eg educate communities about research, collaborate for increased funding, guide government and sponsors). To date, research advocates have collaborated on 444 jobs across 237 unique academic research and drug development projects.

Through practice‐based observation, the Foundation identified gaps and critical issues in patient engagement, such as representativeness of patient populations, a need for harmonized metrics, project management assistance from patient advocacy organizations with expertise in patient engagement and the need to disseminate results. As the Foundation networked with other organizations doing patient engagement, through conference presentations, FDA Patient‐Focused Drug Development meetings or warm introductions, themes in conversations in patient engagement emerged that aligned with Foundation findings. Ultimately, coalitions were formed, or projects were developed within existing coalitions, to further explore and address these themes. The Foundation worked with these organizations (Patient‐Focused Medicines Development, Clinical Trials Transformation Initiative) to survey industry sponsors and patient organizations about perceived gaps in patient engagement and desired tools and resources.

Parkinson's Foundation also conducted a survey among both United States and United Kingdom Parkinson's researchers in 2017 and found that researchers were unsure of how to conduct patient engagement, unsure of what their roles should be and looked to patient advocacy organizations to assist them with patient engagement projects. In recognition and reinforcement of the need to develop a resource that could clearly define steps and party involvement, Parkinson's Foundation has developed a framework for practicing patient engagement when partnering with organizations within the academic research and drug development sectors. As discussed, this approach was developed over the course of several years through assessing the literature, adapting disease‐specific practices to be relevant and generalizable across patient engagement projects and acquiring feedback from researchers, patient advocacy coalitions and people with Parkinson's. Through work on 237 projects, Parkinson's Foundation identified several domains that encompass the scope of the patient engagement process and key project execution components. The six features presented here were found to be the most critical among them for framing and sustaining positive, impactful patient engagement. These identified components can and should be used in conjunction with other identified best practices and measures available to guide the patient engagement process.

This framework includes methods for engagement and metrics that assess both the quality and associated outcomes of patient engagement on the project. These methods include the following:

1. Creation of a scope of work.
2. Establishment of guiding principles.
3. Selection and training of participants.
4. Co‐determination of project metrics.
5. Execution of the project.
6. Dissemination of project findings.

*A detailed case example detailing each step of the framework can be found in Table 1.

**Table 1:** A case example for fostering positive patient engagement practices utilizing framework steps one through six

A Case Example for Fostering Positive Patient Engagement Practices Utilizing Framework Steps One Through Six
Two stakeholder groups, Parkinson's Foundation and an unnamed pharmaceutical company (Company X), utilized a structured collaborative approach to patient engagement in research.
*Step 1:* Over the course of six months, Parkinson's Foundation and Company X worked to create a scope of work. Company X was looking to get feedback on a study protocol for a drug in Phase II clinical trials prior to issuing a protocol amendment. Parkinson's Foundation communicated with the legal, research and accounting teams to determine project expectations. Together, Parkinson's Foundation and Company X decided that a one‐day audio‐recorded focus group with six patient advocates, three Parkinson's Foundation staff and three Company X researchers would be most conducive to eliciting the type of feedback requested. Parkinson's Foundation and Company X together agreed that the project would be completed within three months.
*Step 2:* Of the several principles discussed in Step 2, Parkinson's Foundation worked with Company X to focus on creating a capacity for positive accessible engagement, respect and transparency in communication. To create a capacity for positive accessible engagement, it was agreed upon that the focus group would be hosted at Parkinson's Foundation (a location many advocates were already comfortable and familiar with), all focus group questions would be co‐created over the course of several virtual meetings between Parkinson's Foundation staff and Company X researchers, any materials pertinent to the focus group would be provided to patient advocates in advance, and the focus group would be facilitated by Parkinson's Foundation staff. To promote respect, it was agreed upon that all focus group attendees would be addressed by first name only (no titles or degrees), patient advocates could ask questions to researchers about the study, and at the end of the focus group, all three researchers would share something they had learned throughout the course of the day. Lastly, to promote transparency in communication, it was agreed upon that all materials would be presented at an appropriate health literacy level, Company X would provide as much detail as possible about the study and investigational drug, and any protocol amendments attributed to the focus group would be shared back with the patient advocates within six months after the project execution stage.
*Step 3:* Parkinson's Foundation identified ten patient advocates that met the criteria Company X had requested and shared anonymized patient advocate profiles with Company X. Company X then selected the six patient advocates they preferred to collaborate with. Through a virtual group call, Parkinson's Foundation staff then worked with the patient advocates to review research content that would be relevant to the focus group. Background information and materials were also provided to patient advocates in advance of the focus group. Parkinson's Foundation also worked with Company X researchers to generate questions at an appropriate health literacy level that could capture patient sentiments and feelings while informing the clinical trial protocol. Company X then adjusted their presentations accordingly.
*Step 4:* Parkinson's Foundation worked with Company X to determine a plan to assess how feedback influenced protocol decisions for both study drugs. It was decided that quality metrics would be assessed through follow‐up phone calls and surveys assessing satisfaction and perceived quality of contribution to the project. Due to limitations in timing, it was decided that outcomes would be measured through whether protocol amendments were made as a result of feedback provided at the focus group.
*Step 5:* Six patient advocates, three Parkinson's Foundation staff and three Company X researchers attended a one‐day, audio‐recorded, in‐person focus group. The day was broken into six separate sessions: five of which were facilitated by Parkinson's Foundation staff and one of which was a presentation facilitated by a Company X researcher. All questions and talking points were scripted. Patient advocates participated in a variety of activities to share feedback including speaking, flip chart activities, diagrams and patient demonstrations. Breakfast and lunch were provided. At the end of the focus group, Company X researchers shared what they had learned from patient advocates that day.
*Step 6:* Parkinson's Foundation analysed all audio and drafted a report detailing topics discussed throughout the day. Parkinson's Foundation also identified eight recommendations for clinical trial improvement based upon themes from focus group discussions. Company X then followed up with patient advocates within six months of project execution to share three protocol changes that occurred as a result of patient advocate feedback. Results of this project were shared in two co‐authored poster publications at the International Conference on Alzheimer's and Parkinson's Diseases and the International Congress of Parkinson's Disease and Movement Disorders. Two additional patient engagement speaking opportunities with Company X later occurred as a result of the positive project experiences between patient advocates, Company X researchers and Parkinson's Foundation.

*1. Creation of a scope of work.* Parkinson's Foundation works with research organizations and pharmaceutical companies to build a scope of work which incorporates patient engagement across several stages of research. Drafting a scope of work at the beginning of any project allows for project expectations, feedback, communication and timelines to be determined early and acts as a guide to hold all involved parties accountable to the identified benchmarks.^14^, ^15^, ^16^, ^17^, ^18^, ^19^, ^20^ Parkinson's Foundation communicates with several parties within a research organization or pharmaceutical company (legal and compliance, research and development, coordinators, principle investigators, etc) to ensure clarity in roles and expectations.

Depending on the depth of the project and anticipated timeline, a scope of work can include patient engagement at one particular phase of research, or it can identify several points and stages of engagement over the course of several years. Working with an experienced patient advocacy organization allows researchers, academic institutions and pharmaceutical companies to explore a variety of patient engagement practices and determine which practices ensure that research is relevant and correctly captures community needs.^21^

*2. Establishment of guiding principles.* Patient‐Focused Medicines Development, an organization that works to integrate patient input throughout the lifecycle of medical research has developed the Patient Engagement Quality Guidance.^22^ This guidance, which Parkinson's Foundation contributed to, includes seven quality standards to identify and apply for purposeful engagement. These seven criteria include the following: shared purpose, respect and accessibility, representativeness of stakeholders, roles and responsibilities, capacity for engagement, transparency in communication and documentation, and continuity and sustainability.^23^ Several of these principles are further elaborated in other steps of the process described in this article. Adopting patient engagement guidelines within research organizations and pharmaceutical companies at the start of the project allows for an expectation to be set that all parties, including patient advocates, will have equal decision making throughout the period of project collaboration.^14^, ^15^, ^16^, ^21^, ^23^, ^24^ Creating transparency about the starting point and finish line for the patient engagement project, fosters a shared vision for all parties, including researchers and pharmaceutical staff, Parkinson's Foundation staff, and patient advocates.

Setting guidelines most often involves Parkinson's Foundation staff, researchers and pharmaceutical staff. It entails discussions about patient engagement project expectations and teasing out capacity for receiving and revising research plans as a result of patient feedback. Patient advocates should be brought in to confer and elaborate on the guiding principles at this stage, which is a model Parkinson's Foundation is working towards as our patient engagement methods evolve and partners become more comfortable with this idea.

*3. Selection and training of participants.* Selecting representative patient advocates is a critical step to ensure positive patient engagement practices.^9^, ^24^ Parkinson's Foundation works with researchers and pharmaceutical staff to identify qualities, including demographic and disease characteristics, that influence and benefit input relative to the patient engagement project. This selection process ensures that a variety of experiences and opinions are shared throughout the project.

For patient engagement to be effective and beneficial to both patient advocates and researchers or pharmaceutical staff, additional time to train both parties in the execution of patient engagement is often required and should not be overlooked when designing a patient engagement plan.^17^, ^18^, ^24^, ^25^, ^26^ It is important that all parties have access to resources and terminology that will promote informative bi‐directional conversations.^17^, ^24^ Parkinson's Foundation works with researchers and pharmaceutical staff to address their organization's level of preparedness to collaborate with patient advocates, and identifies the training and resources needed to foster positive engagement practices. This includes training researchers and pharmaceutical staff in maximizing communication with research advocates by providing guidance on appropriate terminology and applying methods to develop and ask patient advocates the right questions.

During this step, Parkinson's Foundation also works to identify the necessary training for patient advocates. Although all Parkinson's Foundation patient advocates have been trained in the research process, advocates are also briefed on project‐specific terminology and communication styles. Patient advocates are trained on scientific background material relevant to the project, how to prepare for project communications, and how to feel comfortable speaking up in a group setting whether in‐person or on a call. People with Parkinson's disease may experience anxiety or cognitive decline, including executive function, multitasking and decision‐making challenges, so it is important to prepare advocates in advance and with as much clarity as possible.

By identifying training needs and expectations at the beginning of the project, all materials can be adequately prepared to help manage the engagement process. Successful patient engagement is the result of equally informed parties contributing to the creation, design, implementation, analysis and dissemination of a study. Training for all parties is a necessary component to making this happen.

*4. Co‐determination of project metrics.* In conjunction with steps two and three, metrics to capture both the quality and associated outcomes of patient engagement on the project should be identified prior to the execution of patient engagement (Step 5). Patient engagement encompasses a broad spectrum of activities, so it is important that metrics match the project. Parkinson's Foundation staff work with researchers and pharmaceutical staff to identify metrics that can be captured throughout the course of the project and that can help inform future projects. The identified metrics are then shared with patient advocates not involved in the project for feedback about readability of collection tools, community relevance and perceived impact. Assessments are administered at project‐appropriate times (these can include formative, interim and summative metrics). These times may vary project to project, but at minimum, a summative assessment is administered to all involved parties (researchers and pharmaceutical staff and patient advocates).

**Figure 1:**
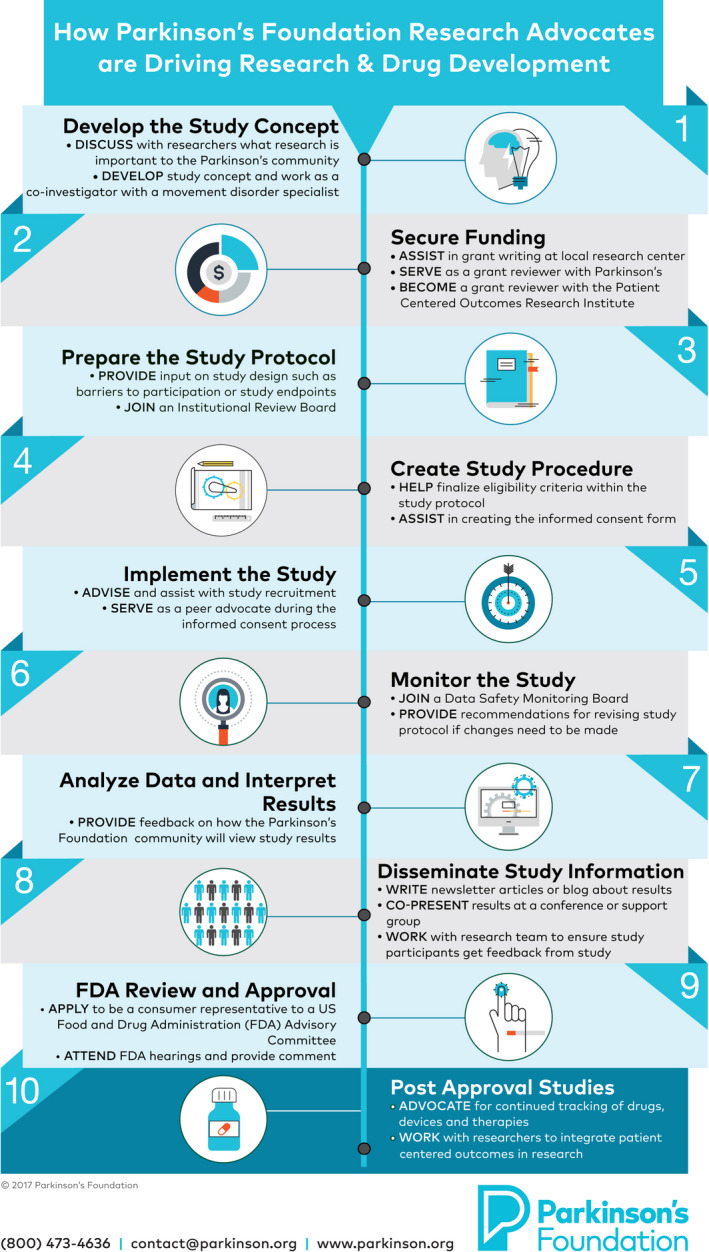
Patient engagement opportunities in drug development

*5. Execution of patient engagement*. Execution of the patient engagement activity encompasses the scope of the interaction process between patient advocates and researchers or pharmaceutical staff (see Figure 1). The length of execution varies by project type and phase, which are determined in the scope of work. Execution can include in‐person meetings, virtual meetings and questionnaires. Parkinson's Foundation works with researchers and pharmaceutical staff to mediate patient engagement activities to create balanced interactions and transparent environments that support the collection of information from patient advocates. With experience, researchers and pharmaceutical staff can communicate with patient advocates independently, but Parkinson's Foundation encourages the use of a third‐party patient engagement expert until a routine for engagement has been established.

*6. Dissemination of project findings.* All parties have something to gain from successful patient engagement, and by practicing the guiding principles of mutual respect, trust and reciprocity (established in Step 2), a culture of patient engagement can be promoted and sustained at an organization.^16^, ^18^, ^19^, ^21^, ^24^, ^26^ To adhere to these principles and align with positive patient engagement practices, all project findings should be reported back to involved parties, including patient advocates. Parkinson's Foundation ensures that patient advocates are included in dissemination by requiring this step to be agreed upon in the scope of work. Parkinson's Foundation also encourages that both researchers and pharmaceutical staff and patient advocates share feedback about engagement satisfaction. This feedback should be considered for and incorporated into future patient engagement projects. Project findings can also be disseminated through conferences, presentations and publications to reinforce identified patient engagement best practices and lessons learned.

## 3 MEASURING THE SUCCESS OF PATIENT ENGAGEMENT

Within this identified patient engagement framework, several metrics can be captured to assess the quality and associated outcomes of patient engagement. Although not exhaustive, Parkinson's Foundation identified a set of metrics to assess patient engagement projects. These metrics were identified over the course of several years through suggestions from FDA, published best practices, collaborations with other patient advocacy organizations and internal piloting across several types of patient engagement projects. After attending and speaking at various meetings including Biotechnology Industry Organization International Convention, Drug Information Association Metrics in Patient‐Centered Drug Development, Patients as Partners and the Clinical Trials Transformation Initiative workshop in collaboration with FDA, 'Enhancing the Incorporation of Patient Perspectives in Clinical Trials', Parkinson's Foundation worked with patient advocates, researchers and pharmaceutical staff to identify metrics that would be realistic to capture within the scope of a patient engagement project. The purpose of these metrics is to identify relevant measures that can communicate the significance of positive patient engagement practices to the research community. After refinement, Parkinson's Foundation identified foundational metrics that can be used to measure the success of a patient engagement project (see Table 2).

**Table 2:** Metrics to capture the quality and outcomes of patient engagement

**Quality metrics include**
*Assessment intended for all involved parties—*
General engagement descriptive statistics Level and form of patient engagement Frequency of patient engagement Satisfaction with patient engagement
Contribution to engagement Perceived quality of contribution compared with project expectations
Training for engagement Preparation and comfort with terminology, communication or interactions Level of support provided compared with support required in the project
Transparency of engagement Perceived maintenance of trust, transparency and bi‐directional communication
*Assessment intended for patient advocates only—*
Representativeness of engagement Representativeness of patients involved in the project
**Outcomes metrics include:**
*Outcomes most often captured and reported by stakeholders—*
Research relevance to disease‐specific topic Research priority or focus shift Reported change in quality of life Selection of relevant patient‐reported outcomes (PRO) tools Clinical trial cost (secondary outcome if met) Time to complete clinical trial (secondary outcome if met)
Alignment to patient preferences Changes made to mode of administration/delivery Frequency of trial visits Number of protocol amendments Length of clinical trial appointment
Patient comprehension of trial expectations and resources Reliable reporting of PRO tools Time to availability of plain language summaries Perceived clarity of trial communications Clinical trial participant satisfaction (secondary outcome if met) Screen fail rates (secondary outcome if met)
Recruitment and retention Barriers to participation in research Clinical trial retention Incomplete data points Length of trial recruitment Clinical trial inquiries
Representativeness of study participants Representation of diverse groups in clinical trial
Application of patient engagement Creation of organizational standards for patient engagement Occurrence of patient engagement practices within the organization

### 3.1 Quality of patient engagement

Within a patient engagement project, the quality of patient engagement should be captured through metrics that assess project performance. These metrics inform whether all parties executed the framework steps and planned patient engagement practices successfully. Each party receives questions specific to their attitudes, assigned roles and project expectations. Parkinson's Foundation leads the collection and analysis of quality metrics; however, the party assigned to this role (most often assigned in the scope of work) will depend on that party's capacity to design, administer and analyse metrics for each of the involved parties in an unbiased manner.

Areas of quality to assess include the following: form of patient engagement and perceived satisfaction by all involved parties, representativeness of patients (as defined by researchers, pharmaceutical staff or patient advocacy organizations in the selection and training of participants), perceived quality of contribution by all involved parties during the execution of patient engagement, quality of training (often provided by patient advocacy organizations) and perceived project transparency by all involved parties. Parkinson's Foundation often assesses patients and stakeholders involved in these areas in the form of online questionnaires and frequency statistics (See Table 3 for sample survey questions). These findings are then reported back to all involved parties for the purpose of informing future patient engagement projects. Quality findings are also shared publicly through publications and conferences.

**Table 3:** Sample quality assessment survey questions

Assessment questions for patient advocates	Assessment questions for researchers and pharmaceutical staff
How many times was your feedback requested throughout this patient engagement project? () Once () Two to three times () Four to five times () More than five times	Did you/your team make an effort to respect and incorporate the opinions of people with Parkinson's in discussions with your team and Parkinson's Foundation staff for this project? ( ) Yes ( ) No ( ) Unsure
Do you feel that your engagement positively impacted this project? ( ) Yes ( ) No ( ) Unsure Please explain.	Did you feel that you had an adequate understanding of your research team's expected contributions to promote patient engagement in this project? ( ) Yes ( ) No ( ) Unsure
Did you feel comfortable sharing your opinion with researchers or Parkinson's Foundation staff for this project? ( ) Yes ( ) No ( ) Unsure	Did you feel that you/your research team was adequately supported to practice patient engagement by Parkinson's Foundation staff throughout the duration of this project? () Strongly supported () Moderately supported () Somewhat supported () Not supported
Did you feel comfortable with the technical level and terminology used during discussions with researchers or Parkinson's Foundation staff for this project? ( ) Yes ( ) No ( ) Unsure	Did patients have the power to contribute towards decisions about research for this patient engagement project? ( ) Yes ( ) No ( ) Unsure

### 3.2 Outcomes of patient engagement

Within a project, metrics should be captured to identify changes made as a result of patient engagement. This can be more difficult to assess as it may take years for research to be completed and, as a result, the outcomes of the related engagement to be determined. Desired change should be discussed at the beginning of a new project, and realistic methods to measure change should be agreed upon by several parties within a research organization (legal and compliance, research and development, health economics and outcomes research, etc). A plan should be developed to capture these metrics within a realistic timeline because they are critical when communicating the value of patient engagement and contributions to research. Parkinson's Foundation works with stakeholder parties to identify which measures of change they intend to capture and the timeline with which to do so. These outcomes expectations are, at minimum, discussed but often included in the scope of work, and metrics are drafted by Parkinson's Foundation. After the allotted time, Parkinson's Foundation follows up with the researchers, academic institution or pharmaceutical company to enquire about the identified outcomes. Outcomes are typically collected internally by the academic institutions or pharmaceutical company. Internal collection reduces barriers around company confidentiality policies or access to staff. The research teams report outcomes back to the Parkinson's Foundation, and the Parkinson's Foundation then works with stakeholders to communicate these findings to patient advocates involved in the project.

Outcomes metrics can vary widely depending on the project. A few outcomes areas to assess could include the following: research relevance to a disease‐specific topic, alignment with patient preferences (mode of administration, frequency of visits, perceived impact of improvement on quality of life, etc), clarity of trial communications, patient comprehension of trial expectations or patient‐reported outcomes tools, recruitment and retention, representativeness of study participants, and organizational application and sustainability of patient engagement. Parkinson's Foundation captures these metrics through follow‐up conversations and questionnaires with stakeholders, as determined in the scope of work. These findings are reported back to patient advocates and are further shared publicly through publications and conferences.

## 4 FUTURE OPPORTUNITIES

The practice of patient engagement has increased in the past decade and has slowly been utilized to support the success of global clinical trials. Patient engagement has the potential to positively impact research; however, it is not currently standard practice to include and report on patient engagement.^8^ Several researchers have incorporated patient engagement practices into their work, but many have only identified metrics that assess reach and count.^7^ The limited evidence that exists about the impact of patient engagement is not yet compelling enough to shift the norm. This framework was created, both to provide a route to ensure that all parties are provided a space to contribute meaningful input and to capture the quality and outcomes of patient engagement in academic research and drug development. In the last several years, Parkinson's Foundation has worked with researchers, academia, industry and the patient community, to develop and refine this framework. This framework should be used in conjunction with other identified best practices and measures available (previously mentioned) to guide the patient engagement process. By creating a common process for capturing outcomes metrics, patient engagement practices can be assessed for effectiveness and refined when necessary. Impactful methods can then be identified and publicly shared. With the support of identified evidence‐based practices, the inclusion of patient engagement in research can become a common practice. Positive and evidence‐based practices can enhance transparency and validate patient engagement in research, which can better address community needs and priorities, utilize stronger patient‐reported outcomes and meaningful endpoints, reduce study burden, and enhance recruitment and retention.^27^ By improving patient engagement project metrics, trials can be more efficiently designed to capture the needs of and ultimately benefit the patient community.

## CONFLICT OF INTEREST

Jori Fleisher has received research support from NIH/NINDS, CurePSP, Biogen, Joyce DeMoose & George Harvey (private philanthropy). Jori Fleisher is a consultant to UCB. Jori Fleisher has received royalties from UpToDate. No conflicts of interest are reported for all other authors.

## Data Availability

Data sharing is not applicable to this article as no new data were created or analysed in this study.
