# Erratum

**DOI:** 10.1111/hex.13128

**Published:** 2020-09-16

The Letter to the Editor by Riggare et al^1^ in response to the Viewpoint Article by Feeney et al^2^ was published without the Journal informing the authors of the Viewpoint Article that the Letter to the Editor was being published nor gave the authors the chance to respond at the same time. The Letter to the Editor also mentioned that there was no patient advocate included on their paper which is incorrect, as Lisa Cone (who has Parkinson’s Disease) co‐authored the Viewpoint Article.

The Editorial Office apologizes for these errors and has revised the process for dealing with letters to the Journal.
